# Dynein Heavy Chain, Encoded by Two Genes in Agaricomycetes, Is Required for Nuclear Migration in *Schizophyllum commune*

**DOI:** 10.1371/journal.pone.0135616

**Published:** 2015-08-18

**Authors:** Melanie Brunsch, Daniela Schubert, Matthias Gube, Christiane Ring, Lisa Hanisch, Jörg Linde, Katrin Krause, Erika Kothe

**Affiliations:** 1 Institute of Microbiology, Microbial Communication, Friedrich Schiller University, Neugasse 25, 07743, Jena, Germany; 2 Research Group Systems Biology and Bioinformatics, Leibniz-Institute for Natural Product Research and Infection Biology–Hans-Knöll-Institute, Beutenbergstraße 11a, 07745, Jena, Germany; Georg-August-University of Göttingen Institute of Microbiology & Genetics, GERMANY

## Abstract

The white-rot fungus *Schizophyllum commune* (Agaricomycetes) was used to study the cell biology of microtubular trafficking during mating interactions, when the two partners exchange nuclei, which are transported along microtubule tracks. For this transport activity, the motor protein dynein is required. In *S*. *commune*, the dynein heavy chain is encoded in two parts by two separate genes, *dhc1* and *dhc2*. The N-terminal protein Dhc1 supplies the dimerization domain, while Dhc2 encodes the motor machinery and the microtubule binding domain. This split motor protein is unique to Basidiomycota, where three different sequence patterns suggest independent split events during evolution. To investigate the function of the dynein heavy chain, the gene *dhc1* and the motor domain in *dhc2* were deleted. Both resulting mutants were viable, but revealed phenotypes in hyphal growth morphology and mating behavior as well as in sexual development. Viability of strain Δ*dhc2* is due to the higher expression of kinesin-2 and kinesin-14, which was proven *via* RNA sequencing.

## Introduction

The split gill fungus *Schizophyllum commune* (Agaricomycotina) is a mushroom forming fungus with world-wide occurrence [1]. It has been used as a model organism for molecular and cell biology due to its easy handling and the ability to pass the entire life cycle within 14 days under laboratory conditions [2]. Transformation and gene knock-out have been established [3] including facilitation of homologous recombination using a *ku80* deletion strain [4]. The available genome sequence allows transcriptome and proteome analyses [5]. Thus, it provides an excellent background for molecular studies, which can draw on a multitude of earlier genetic studies including the tetrapolar mating system, nuclear migration studies, or micromorphology [6–10].

In the life cycle of *S*. *commune*, homo-dikaryotic meiospores germinate and monokaryotic mycelia with one nucleus per cell are formed. When two compatible monokaryons of different specificity of mating pathways *A* (with *A* genes encoding homeodomain transcription factors) and *B* (the genes coding for a multispecific pheromone/receptor system) fuse, a fertile dikaryon is established [3]. During the change from monokaryon to a functional dikaryon, the reciprocal exchange of nuclei between the mating partners is essential [2, 11]. This process has been clocked for *S*. *commune* with 25–90 μm/sec in dikaryotization experiments [12].

The major minus-end directed microtubule-dependent motor protein is the dynein complex. In filamentous fungi like *Aspergillus nidulans* and *Ustilago maydis*, dynein is accumulated at the plus-end of microtubules, which is necessary for the full function of the complex [13, 14]. Dynein is required for vesicle transport, nuclear migration and positioning as well as spindle assembly during mitosis and meiosis [15–18]. Since all these processes are involved in nuclear migration, the minus-end directed motor protein dynein is expected to be a major driver for nuclear exchange during fungal mating [19]. All dynein complexes consist of different homologous subunits: two identical heavy chains (approx. 500 kDa), several intermediate chains (60–150 kDa), light intermediate chains (~ 60 kDa) and light chains (< 60 kDa) [20–23]. The dynein heavy chain (~ 4600 aa) contains an approximately 1200 aa N-terminal region which forms the tail of the heavy chain. This region contains the dimerization domain and binding sites for accessory proteins. The C-terminal region of approximately 3400 aa encodes the motor machinery [24] which encompasses six AAA-modules (ATPases associated with cellular activities). Each of the first four modules contains a Walker A motif (GXXXXGKT/S) necessary for nucleotide binding [25]. Between the fourth and the fifth module, the so called B-link is located. This structure connects the dynein molecule to microtubules [26–29].

In the corn smut fungus *Ustilago maydis*, the dynein heavy chain was found to be encoded by two separate genes, *dyn1* and *dyn2*. They are split within the fourth AAA-module, the knock-out of either of the two genes is lethal [30]. This finding prompted us to look into the mushroom forming homobasidiomycete *S*. *commune*. Indeed, dynein heavy chain is encoded by two genes in this fungus as well. However, the gene organization strikingly differs from that of *U*. *maydis*. By comparing other other basidiomycetes, we could identify three different types of split dynein heavy chain proteins indicating independent events in evolution, with the basal *Cryptococcus neoformans* containing a normal, unipartite dynein heavy chain. To investigate the function in homobasidiomycetes, we deleted *dhc1* and the motor domain in the gene *dhc2*. The knock-out of either gene was viable, allowing us to functionally correlate Dhc2 to nuclear migration and mating response.

## Materials and Methods

### Strains and growth conditions

Strains of *S*. *commune* (Table 1) were grown on complex yeast medium (CYM) [31] with the addition of 4 mM tryptophane for *trp1* ^-^ strains at 30°C for 5 to 7 days.

**Table 1:** *S*. *commune* wildtype and mutant strains used in this study.

strain	mating type	characterization	Origin
12–43	*A*_3,5_ *B*_2,2_	*ura* ^*-*^	Jena Microbial Resource Collection
E6	*A*_4,6_ *B*_2,1_	*ura* ^*-*^, *trp* ^*-*^	Jena Microbial Resource Collection
4–40	*A*_4,6_ *B*_1,1_		Jena Microbial Resource Collection
4–39	*A*_1,1_ *B*_3,2_		Jena Microbial Resource Collection
T2	*A*_4,1_ *B*_3,2_	*ura* ^*-*^, *trp* ^*-*^	Jena Microbial Resource Collection
T41	*A*_4,7_ *B*_8,4_		Jena Microbial Resource Collection
∆*dhc1*	*A*_3,5_ *B*_2,2_	deletion in *dhc1*	this study
∆*dhc2*	*A*_3,5_ *B*_2,2_	deletion in *dhc2*	this study

### Identification of dhc1 and dhc2

Dynein heavy chain sequences of different fungi were aligned with the Clustal algorithm as implemented in DNASTAR (version 4.03) and well conserved regions were used as templates to develop degenerated oligonucleotide primers listed in Table 2. DNA isolation was performed using the procedure of Wendland *et al*. [32]. PCR amplification was used to clone *dhc1* and *dhc2* from genomic and cDNA using the genomic sequence (http://genome.jgi-psf.org/Schco1/Schco1.home.html). Hybridization was performed as describen [33].

**Table 2:** Oligonucleotides used.

	sequence (5´-3´)
dhc1AaXbaI	AGT CTC TAG AGC CAC ACC GAA TCG CCA GCA AGT C
dhc1AbXbaI	CCA TTC TAG AGG GGG AAG CAG CGC GAG CAG GTA
dhc1Ba	ACG CGC ATA GGG GCA CCG ACA A
dhc1Bb	GCA AGC GCC ATC CCC AGC AGT
Dyn1*	CCNGCNGGNACNGGNAARACNGA
Dyn3*	ARNCCRAARTCRTARTG
Dyn4*	TGYTTYGAYGARTTYAA
dyn2-1up*	CAGCAGAAYGTBGABATHCC
dyn2-8low*	GCCCAKATKATBGCBCC
dhc*Cla*I	CCATCGATGCACCAGCTCCGTGAT
dhc*Kpn*I	GGGGTACCGAGTGGGAGACGACGC
E6-1rev	GCTTCGCCTCCTTGATGA
dyn2-2for	CATGCCTTGGTCTTCTTCTTG
kin2-1fw	AACTGCACGCTTTTCGCTTACG
kin2-1rev	CCGGCGTTAGAGGATGGG
kin14start-for	ATGGACGCCGAGCGCGCCAA
kin14start-rev	CTACTTCACTACCCTCCTCGCCG

*degenerated primers

### Phylogenetic analyses

Protein sequence data were obtained from public databases NCBI (www.ncbi.nlm.nih.gov), JGI (www.jgi.doe.gov), Broad institute (www.broadinstitute.org), MIPS (http://mips.gsf.de), INRA (http://mycor.nancy.inra.fr) and from J. Mondego (LGE lab, UNICAMP, Brazil) (S1 File, Table A). Dynein heavy chain protein sequences of representatives with split DHC genes (*i*.*e*. Agaricomycetes, Dacryomycetes, Wallemiomycetes and Ustilaginomycotina) were concatenated, allowing for simultanous analysis of all samples. The sequences were aligned using the E-INS-I option of MAFFT v6 [34] which assumes multiple conserved domains and long gaps. For control of alignment quality and adjustment, BIOEDIT v7.0.9.0 [35] was used.

Phylogenetic reconstruction was performed at the Bioportal of the University of Oslo, Norway (http://www.bioportal.uio.no) and the CIPRES science gateway [36], using MrBayes v3.1.2 [37, 38], and RAxML v.7.2.6 [39, 40] for comparison. For Bayesian analysis, the mixed model option with four gamma categories was implemented, allowing for model jumping during the analysis. For all datasets, two runs with each 2,000,000 generations in four chains were performed; sampling every 100 generations, and with a burn-in of 25 percent. Results were evaluated with Tracer v1.5 [41]. Both analyses had log likelyhood ESS values above 100. For the RAxML Maximum Likelihood analysis, phylogeny was inferred under the PROTGAMMAGTR model with four gamma categories and branch support through 100 bootstrap replicates. For visualisation of phylogenetic trees, FigTree v1.3.1 [42] was used.

### Microscopy

Immunofluorescence staining was performed after modified protocols from Raju and Dahl [43] and Fischer and Timberlake [44]. S. commune was inoculated on CYM capped with cover slides at 28°C for 3 d. The cover slides were fixed with PME buffer (0.1 m PIPES pH 6.9, 2 mm EGTA, 1 mm MgSO_4_, 1 mm DTE, 0.1 mm GTP) containing 3.7% formaldehyde for 90 minutes, washed with PME buffer three times followed by cell wall degradation with lysing enzyme (Glucanex, Sigma Aldrich, Munich, Germany) for 20 minutes. For permeabilization, Triton X-100 dissolved in PBS was added for 5 minutes. After 5 minutes of blocking with 0.3% milk powder, the first antibody (1:50 ScDHC1 or ScDHC2, BioGenes, Berlin, Germany) was added and incubated over night at 4°C. The first antibody was removed by washing with PBS. After the incubation with the second antibody (T6778; F6258, Sigma Aldrich, Steinheim, Germany) at a concentration of 1:100 and 37°C for 1 h and washing with PBS, embedding medium containing DAPI fluorescence dye (1 μg/ml) was added for mounting. The samples were investigated using an Axioplan 2 (Carl Zeiss AG, Jena, Germany) and Spot Advanced (Version 4.6, Diagnostic Instruments, Sterling Heights). For confocal images, LSM 5 Axio Observer (Carl Zeiss AG, Jena, Germany) and a Plan Apochromat 63x/1.40 Oil DIC M27 objective were used. Images were analyzed with Zen2009 (Carl Zeiss AG, Jena, Germany).

For scanning electron microscopy, mycelium of *S*. *commune* was air dried for one week on a sterile glass slide and additionally incubated in an exsiccator for three days. Samples were fixed on a sample holder and were sputtered with gold (EMI Tech K500). Samples were analyzed (Philips XL 30 ESEM) using Scandium software (version 5.0, analySIS Image Processing Soft, Imaging System GmbH, Münster, Germany).

### Deletion of dhc1 and dhc2

DNA of the wildtype strain 12–43 was isolated [32]. Flanking regions to *dhc1* were amplified with oligonucleotides dhc1AaXbaI and dhc1AbXbaI for the 1585 bp large upstream flank. Analog, the 1699 bp large downstream flank was amplified with the oligonucleotides dhc1Ba and dhc1Bb. The upstream flank was cloned into *Xba*I restriction sites of the cloning vector pChi which already contained the marker gene *uraI*. The marker gene *ura1*, linked to the *tef* promoter of *S*. *commune* [45] was cloned in between the two homologuous flanks, using *Kpn*I/*Cla*I for the 5´flank, *Cla*I for *tef-ura* and *Cla*I/*Bam*HI for cloning of the 3`flank, resulting in vector p∆*dhc2* [46].

The gene cassette of the upstream flank and the marker gene was restricted with *BamH*I and *Not*I and cloned into the appropriate restriction sites of the vector pBluescript II SK. The downstream flank was cloned into *EcoR*I restriction site of pBluescript II SK. This procedure resulted in the deletion vector pΔ*dhc1*. The flanking regions of *dhc2* were obtained by cloning the 4229 bp *Bam*HI/*Cla*I fragment from a sub-library of respective BamHI/ClaI genomic fragments after screening with a probe obtained from clone pD5. For the 5´ area, an 1863 bp fragment amplified with oligonucleotides dhc*Cla*I and dhc*Kpn*I, was cloned in the *Cla*I/*Kpn*I-restriction sites of the cloning vector. Protoplasts of strain 12–43 were transformed according to Munoz-Rivas *et al*. [47]. For transformation, 20 μg plasmid-DNA of pΔ*dhc1* respectively p∆*dhc2* were used. In order to complement for potentially lethal *dhc2* knock-out, macerated mycelium of the compatible wildtype strain E6 was mixed with the transfected protoplasts. DNA of the resulting dikaryons was tested for the deletion of *dhc2* by PCR. Spores of the positive dikaryon were isolated, plated on selective media and monokaryotic strains with the deleted *dhc2* gene were harvested. For *dhc1*, transfected mycelium was plated to selective media as lethality had not been observed with deletion of *dhc2*. Transformants were analyzed by PCR for the successful deletion of *dhc1*.*Transcriptome analyses*

RNA of seven days old, solid cultures of the strains 12–43, E6 and ∆*dhc2* was isolated using RNeasy Plant Mini Kit performing an additional DNAse digestion with RNase-Free DNase Set (both Qiagen, Hilden,Germany). RNA-sequencing was performed by LGC Genomics, Berlin (Germany) with mRNA-based cDNA-libraries constructed from sequencing adaptors ligated to cDNA fragments. The transcriptome was sequenced from two biological and technical replicates each.

Raw data of RNA sequences were mapped against the genome of *S*. *commune* (genome.jgi.doe.gov/Schco2) using the splice junction mapper TopHat (release 1.4.1) [48]. Htseq (www.heber.emgl.de/users/anders/HTSeq/doc/index.html) was used to calculate the number of reads mapped within each gene (raw counts). Normalized (gene length, library size) expression values (RPKM) for all genes were calculated using the statistical software R. For expression differences, the ratios (fold-change) of mean values (wildtype): mean values(∆*dhc2*) were determined. Four different statistical tests, DeSeq [49], EdgeR [50], BaySeq [51] and Noiseq [52] were used to scan for significantly differentially expressed genes (false discovery rate adjusted p-value cutoff 0.01). A gene was defined to be differentially expressed if it was detected by each method.

To validate RNA-sequencing data, quantitative real-time PCR was performed after Erdmann *et al*., 2012. cDNA synthesis was modified by using the QuantiTect Rev. Transcription Kit (Qiagen, Hilden, Germany).

## Results

### Genomic structure of dynein heavy chain encoding genes

Two independent genes coding for the N-terminus and C-terminus of the dynein heavy chain were identified *via* Southern blot analyses in the genome of *S*. *commune*. Both genes, *dhc1* and *dhc2*, are located on scaffold 2 with a distance of 413 kb in opposite reading directions (Fig A in S1 File). No additional sequences with similarity to parts of dynein heavy chain encoding genes of fungi were found in the genome. While *dhc1* is the smaller gene with a size of approximately 5 kb interrupted by 9 introns, *dhc2* is approximately 11 kb and interrupted by 16 introns. Spores of crosses between strains ∆*dhc2* and the wildtype E6 germinated normally. In 89.5% of the mutant strains, the *matA* mating type specificity of the parental strain *A*_*3*,*5*_ was retained, while 90.4% of *dhc2* wildtype spores showed the *matA* specificity *A*_*4*,*6*_ of the mating partner verifying linkage to *matA* with a distance of 586.5 kb between *dhc2* and *Aβ*.

**Fig 1:**
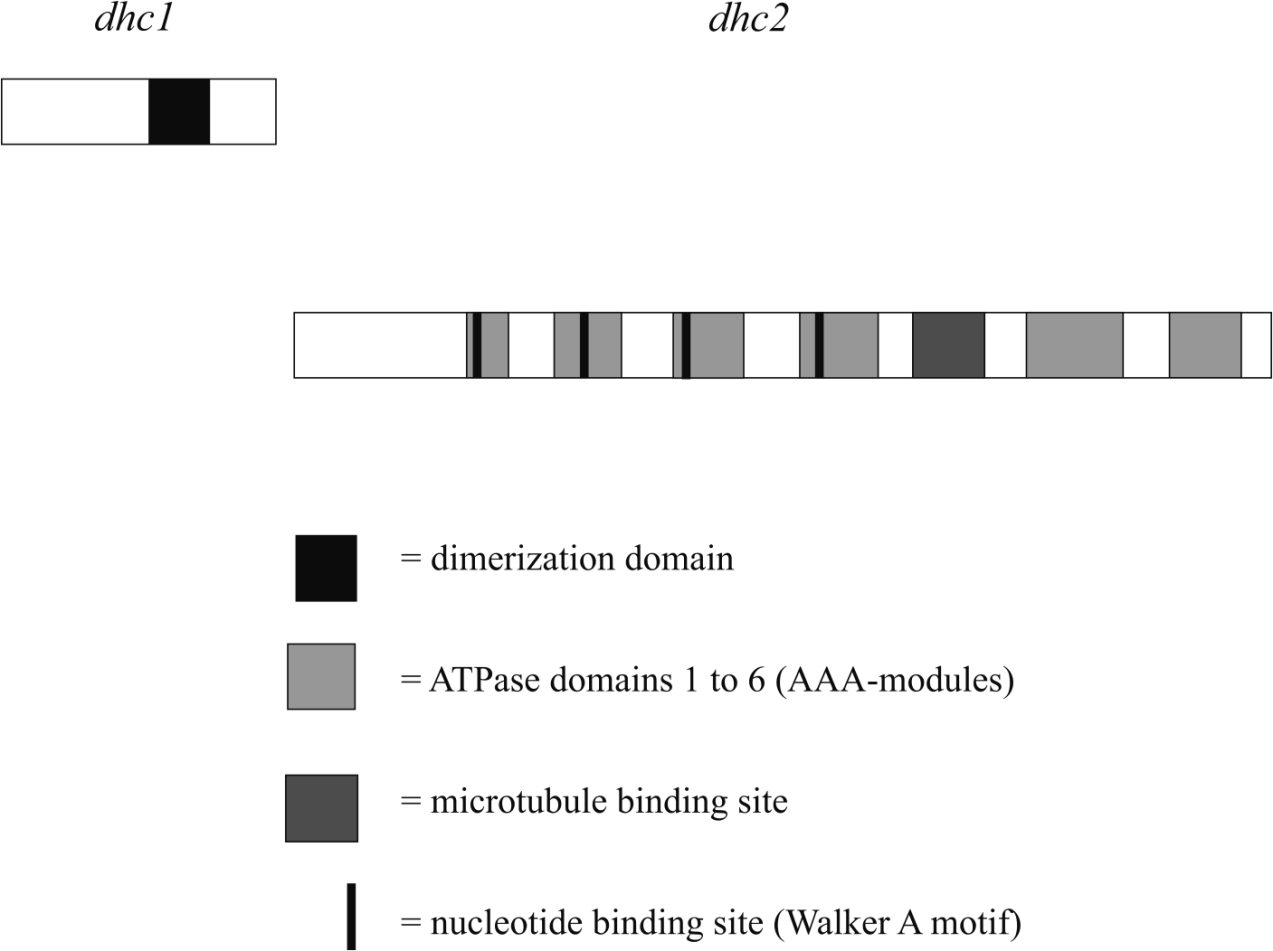
Functional domains in Dhc1 and Dhc2 in *S*. *commune*. The p loops in the four Walker motifs AAA1 (GPAGTGKT) and AAA3 (GPPGSGKT) are identical between *Schizophyllum commune*, the filamentous ascomycete *A*. *nidulans*, the yeast *S*. *cerevisiae* and human. Motifs AAA2 (GPSGSGKT) and AAA4 (GVSGSGKT) are well conserved.

The gene *dhc1* codes for 1180 aa containing a part of the N-terminal tail region, including the typical dynein dimerization domain (576–809 aa). The remaining tail region, as well as the C-terminal region of dynein heavy chain, is encoded by *dhc2* with 3450 aa containing the functional motor machinery. This motor domain is formed by six AAA modules, with the first four modules containing each a highly conserved P-loop structure with a Walker A nucleotide binding motif. Between modules four and five, two α-helical structures bordered by highly conserved proline residues can be found. These coiled-coil regions are known to form the microtubule binding site. Together, the two derived proteins Dhc1 and Dhc2 cover the entire length of a consensus dynein heavy chain (Fig 1).

However, at the split point between both proteins, no additional sequence, for example for protein-protein interaction, is visible. To achieve functionality, however, the two gene products have to be combined to the full dynein complex. We could show that this organization in two independent genes was not specific to the two strains 12–43 used for cloning and H4-8, from which the genome sequence had been derived. PCR with primers E6-1rev and dyn2-2for could amplify the characteristic fragments of the split point containing the promotor of *dhc2* in all analyzed strains (12–43, 4–40, 4–39, E6 and T2).

### The split dynein heavy chain is a unique phenomenon in basidiomycetes

**Fig 2:**
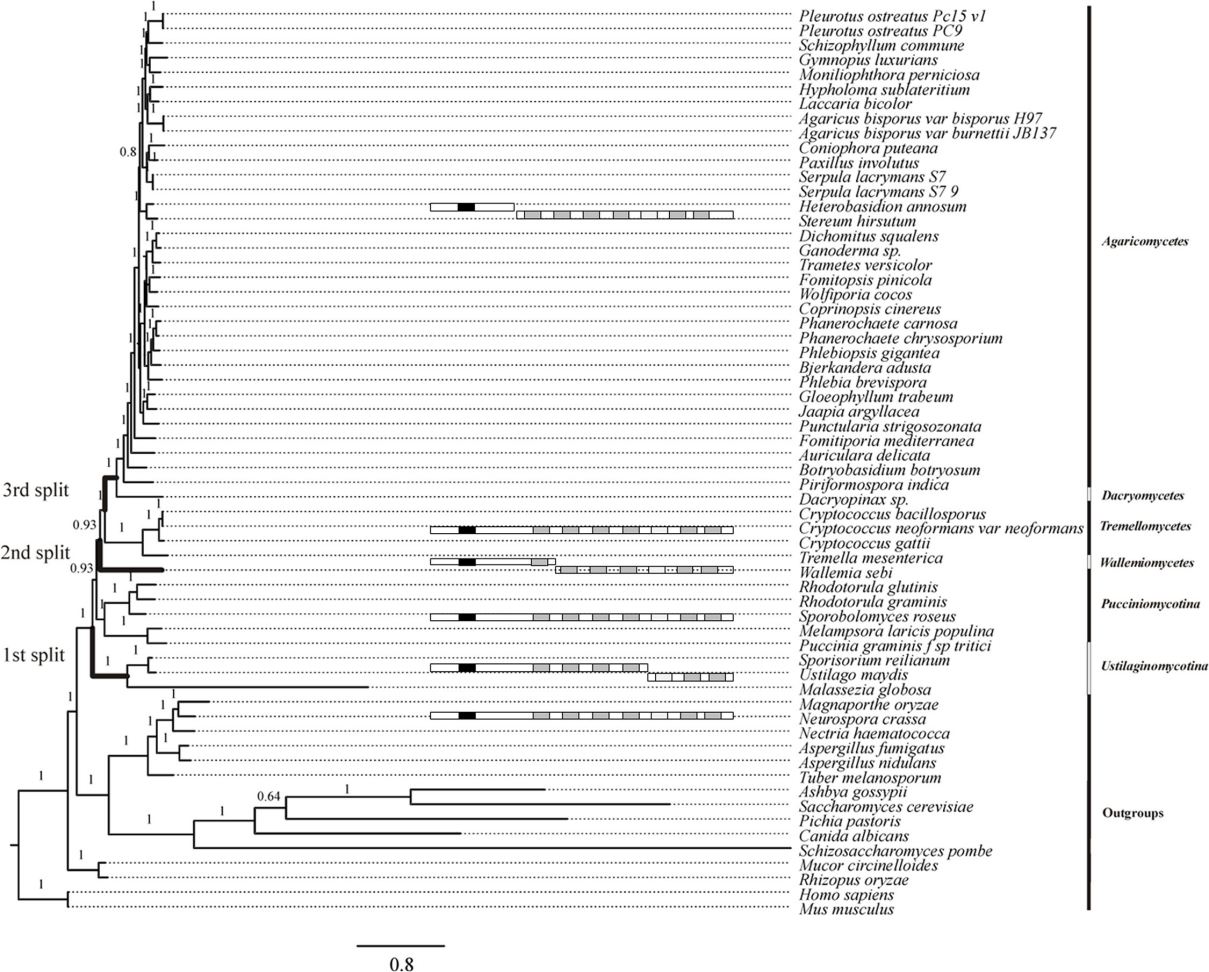
Phylogeny of dynein heavy chain encoding genes. Bayesian posterior probabilities are indicated above corresponding branches. Dynein heavy chain organization is indicated by class or subphylum level.

Of the analyzed dynein heavy chain genes, only Basidiomycota are affected by a division of the dynein heavy chain into two separate proteins, as seen in Bayesian phylogenetic analyses (Fig 2), which was supported by maximum likelihood analyses (data not shown). The split was not found in any other eukaryotic domain, despite the generally high conservation of dynein heavy chain genes from all kingdoms. In comparison with different fungal and most other Eukaryote lineages, the dynein heavy chain genes of Ascomycota yeasts (Saccharomycotina and Taphrinomycotina) show a distinctly accelerated mutation rate, as visualized by the considerably longer branches in the phylogenetic tree. Within Basidiomycota, three splits of the gene are evident, namely in Ustilaginomycotina, in Wallemiomycetes, and in the Agaricomycetes/Dacryomycetes group. With the exception of *Malassezia globosa* (Ustilaginomycotina), conspicuous mutation rate accelerations are not evident. No reversion to the unsplit state was observed, while in the basal Pucciniomycotina and in Tremellomycetes, the dynein heavy chain had remained unipartite. In Ustilaginomycota, a split point is conserved within the fourth AAA module, while the split in Wallemiomycetes occurs after 1979 aa, between the first and the second AAA module. The split point does, in both cases, differ from that found in *S*. *commune*, where it occurs between the dimerization domain and the AAA boxes at aa 1180. This split is unique to Agaricomycetes and Dacrymycetes, and it is highly conserved there (Table 3). This indicates three independent evolutionary events of splitting. In addition to the conserved split point between *dhc1* and *dhc2*, their organization within agaricomycete genomes with respect to the linked *matA* locus seems conserved. Both *dyn1* and *dyn2* genes are located on the same scaffold with distances up to 500 kb and no instance was found, where larger different contigs separate both genes. Also, all *dhc2* homologs are linked to the *matA* locus. However, no synteny is evident in intervening genes, nor in orientation of *dhc1* and *dhc2*.

**Table 3:** Sequence similarities at the split point of Dhc1 and Dhc2 in Agaricomycetes. Similarities at the Dhc1 end are written in bold and italics, and similarities at the start of Dhc2 are in bold and underlined. For comparison, non-agaric fungal dynein heavy chain genes are included to show the basal, fused sequences.

Species	Dhc1	Dhc2
*A*. *bisporus*	***RN***E***LEHQSIEGLVGAPRRF***	MEIFTPS**NSSTAAAVTFITFVQ**
*A*. *delicata*	***RN***E***LEH***H***SIEGLVGAPRRF***	MELIAPN**NSSTAAAVTFITFVQ**
*D*. *squalens*	***RNDLEHQSIEGLVG***P***PRRF***	MELLAPS**NSSTAAAVTFITFVQ**
*C*. *cinerea*	ATTTIYDAFRRRLPVLNDG	MEVRP**N**TIQKYTSYRCSLGVR**TF**K
*F*. *mediterranea*	***R***HE***LE***QH***SIEGL***Q***GAPRRF***	MEVRLEVAFASP**NSSTAAAVTFITFVQ**
*Ganoderma spec*.	***RNDLEHQSIEGLVGAPRRF***	MELLAPS**NSSTAAAVTFITFVQ**
*P*. *chrysosporium*	***R***HE***LEHQSIEGLVGAPRRF***	MSMEVLAPS**NSSTAAAV**Q**FITFVQ**
*P*. *ostreatus*	***RNDLEH***H***SIEGLVG***GV***RRF***	MEAFAS**NSSTAAAV**Q**FITFVQ**
*S*. *commune*	***RNDLEHQSIEGLVGAPRRF***	MPQATASPS**NSSTAAAVTFITFVQ**
*S*. *lacrymans*	***RNDLEH***H***SIEGL***L***G***GT***RRF***	MEAFSPST**SSTAAAVTFITFVQ**
*W*. *cocos*	***RNDLEHQSIEGLVGAPRRF***	MELLTSS**NSSTAAAVTFITFVQ**
*U*. *maydis*	***R***VE***LE***QH***SIEG*** **SSTA**Q**AVTFITFVQ**	
*W*. *sebi*	***R***YE***LE***T***QSIEG*** **SST**VQ**AV**S**FITFVQ**	
*S*. *roseus*	***R***H***DLE***QH***SIE***T**SSTAA**T**VTFITFVQ**	
*N*. *crassa*	***R***K***DLE***G***Q***AMTA**NSTA**E**AV**R**FITIVQ**	
*A*. *fumigatus*	***R***H***DLE***GK***S***LDA**SSTA**H**AV**S**FITIVQ**	

**Fig 3:**
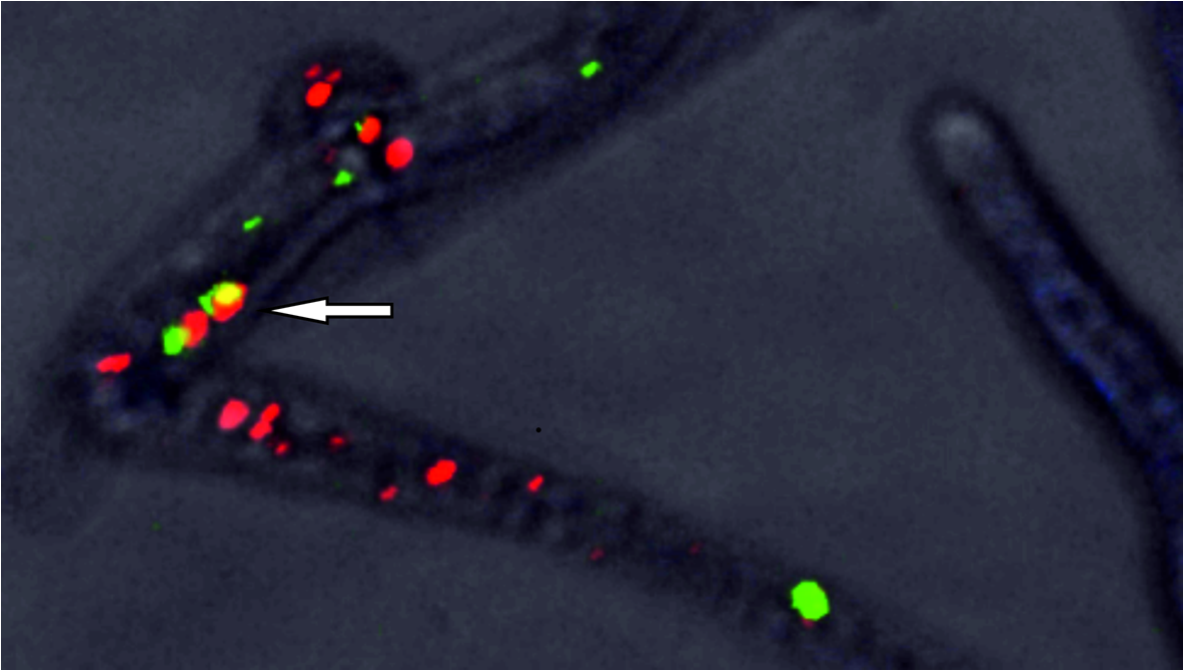
Co-Localization of Dhc1 and Dhc2 in dikaryotic hyphae of *S*. *commune* 12–43. The labeling for Dhc2 is shown in green, Dhc1 in red and DAPI staining visualizes DNA in mitochondria.

Using specific antibodies against heptamers of Dhc1 and Dhc2, both proteins could be detected in the cytoplasm. They were localized close to microtubules, and close to nuclei, with potential co-localization of Dhc1 and Dhc2 (Fig 3). No specific enrichment at hyphal tips for one of the two proteins or in mitotic nuclei could be unequivocally shown (Figs B and C in S1 File).

### Deletion of dynein genes

**Fig 4:**
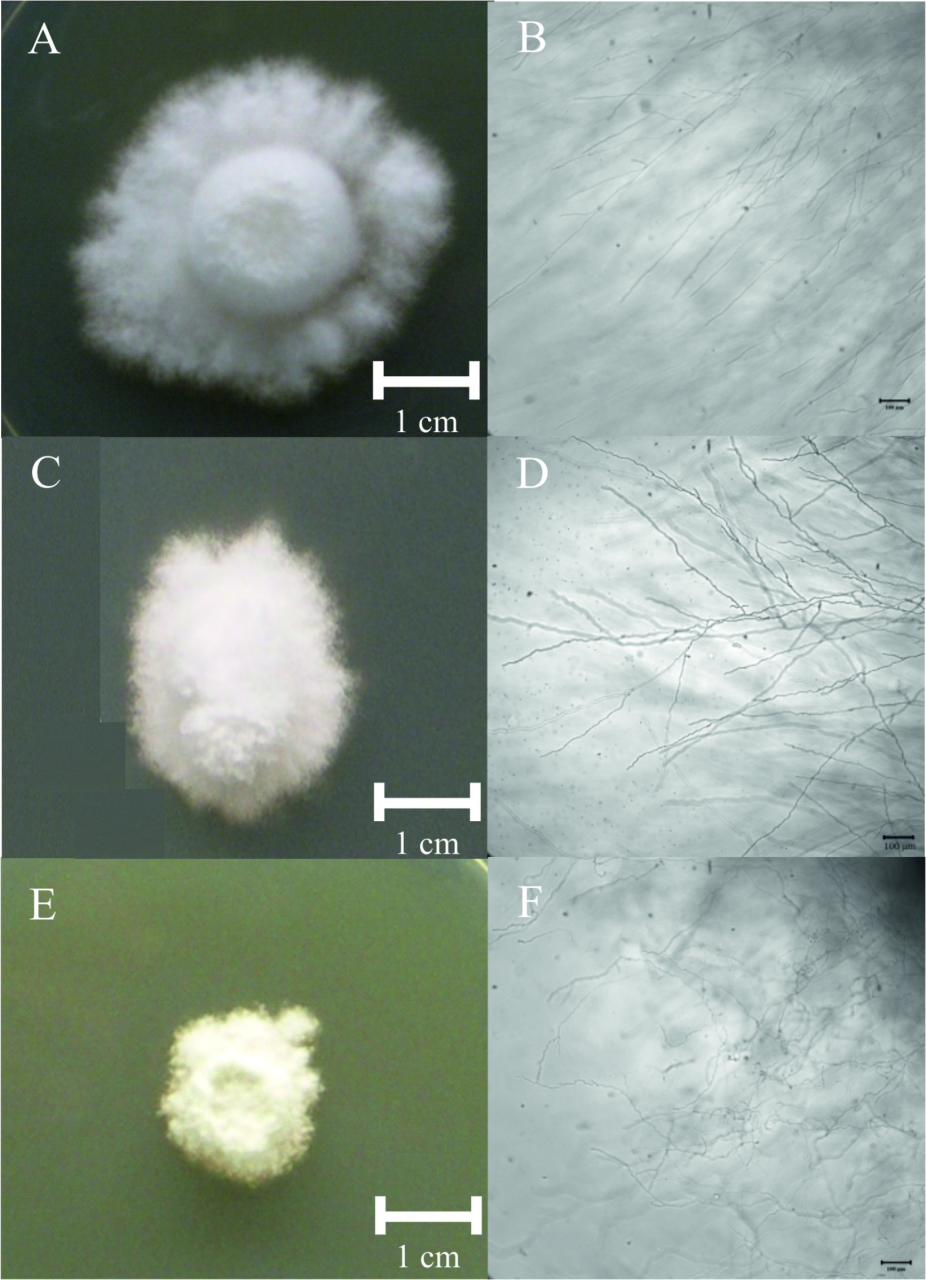
Images of colony structure and hyphal morphology in *S*. *commune* wildtype (upper row), Δ*dhc1* (central row) and ∆*dhc2* (lower row) after 5 days of growth. Phenotypes visible for the deletion mutants were visible with retarded growth, disoriented growth with Δ*dhc1* and deformed hyphae in the *dhc2* deletion strain.

**Fig 5:**
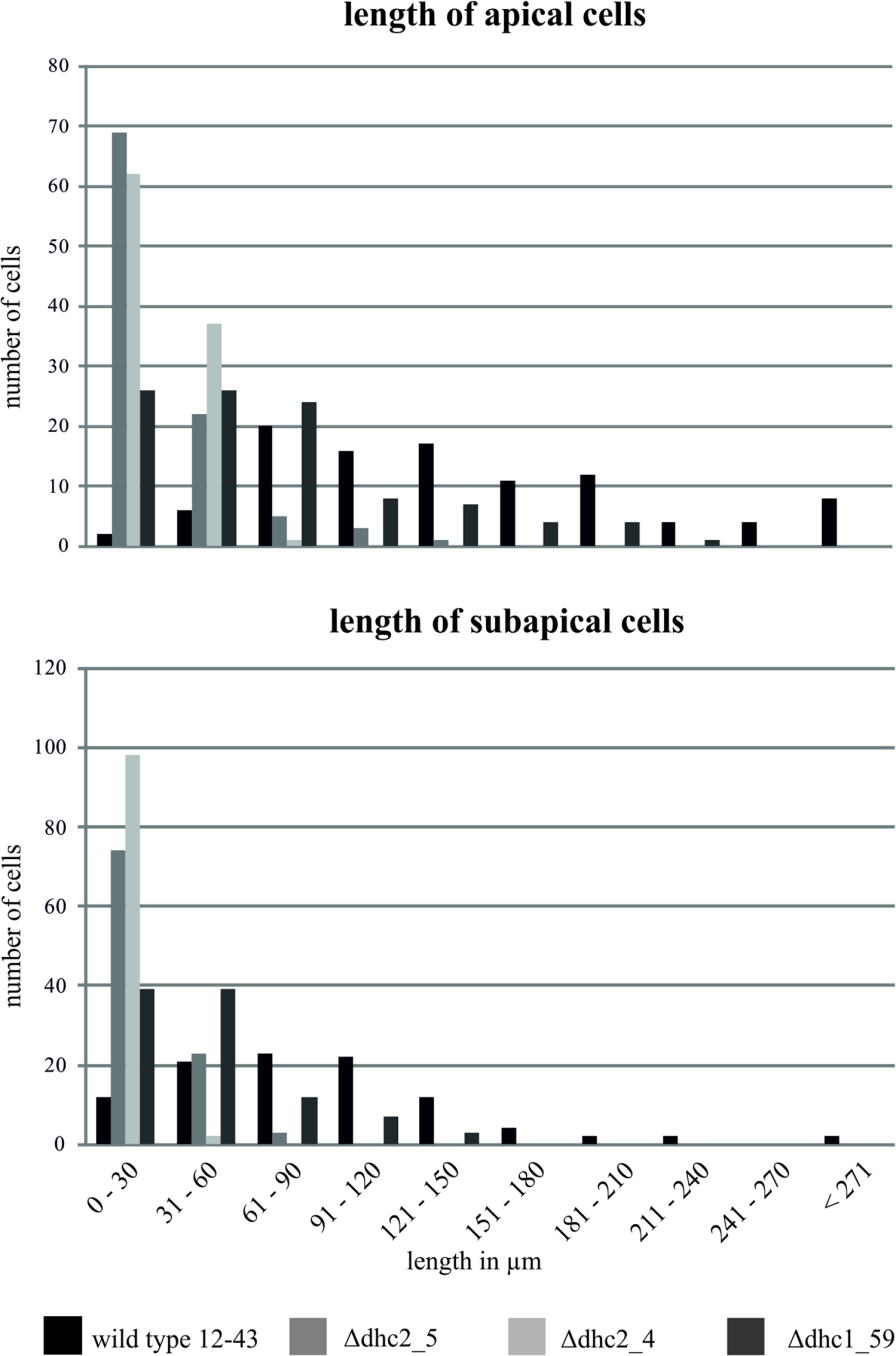
Numerical assessment of phenotypic changes in ∆*dhc* strains. Apical and subapicalcells show a reduced growth; n = 100.

**Fig 6:**
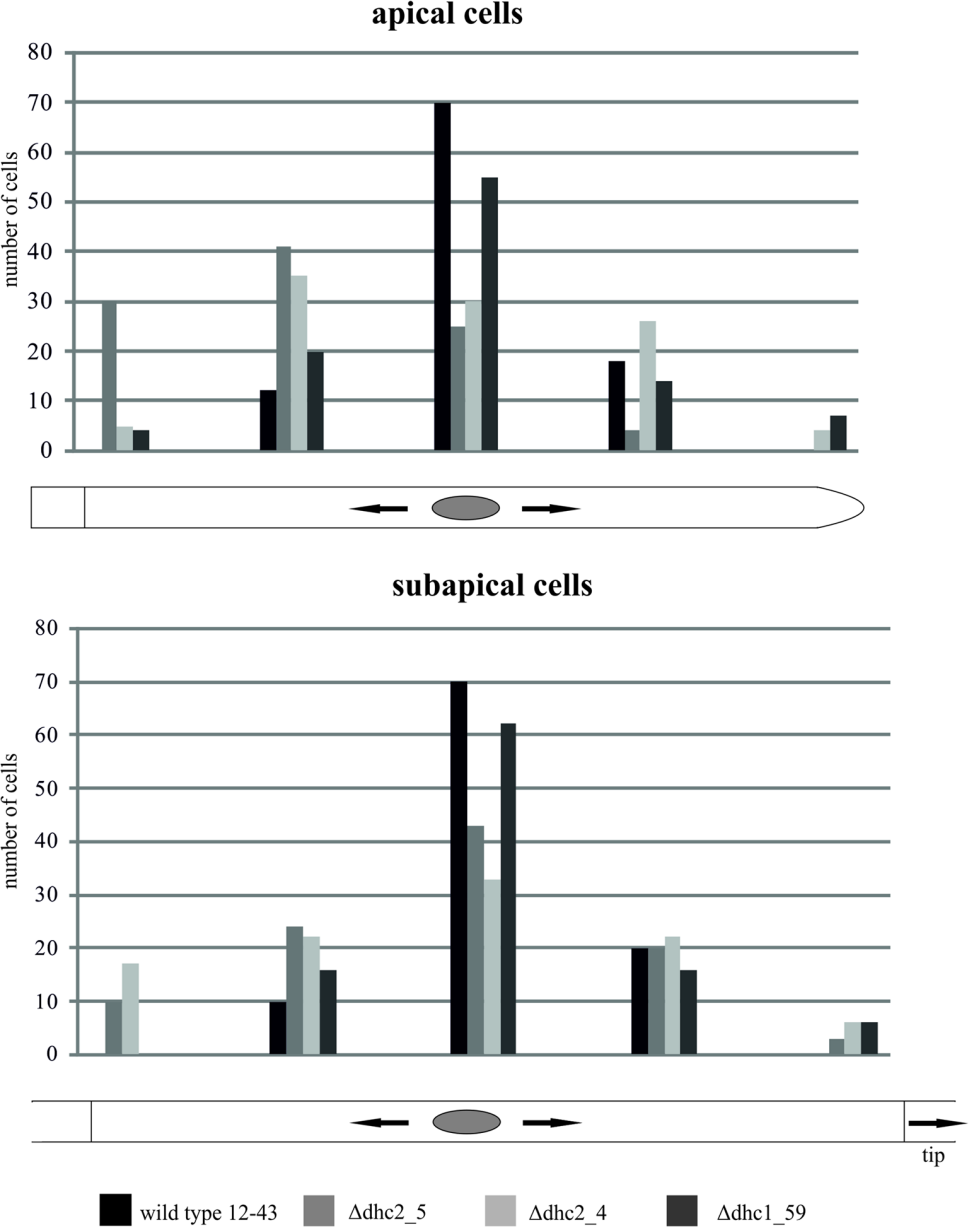
Nuclear distribution in Δ*dhc* strains. Wildtype nuclei are located in the center of a cell, while ∆*dhc* strains feature random distribution (measurements were taken from the septum in apical cells and from the older septum in subapical cells); n = 100.

**Fig 7:**
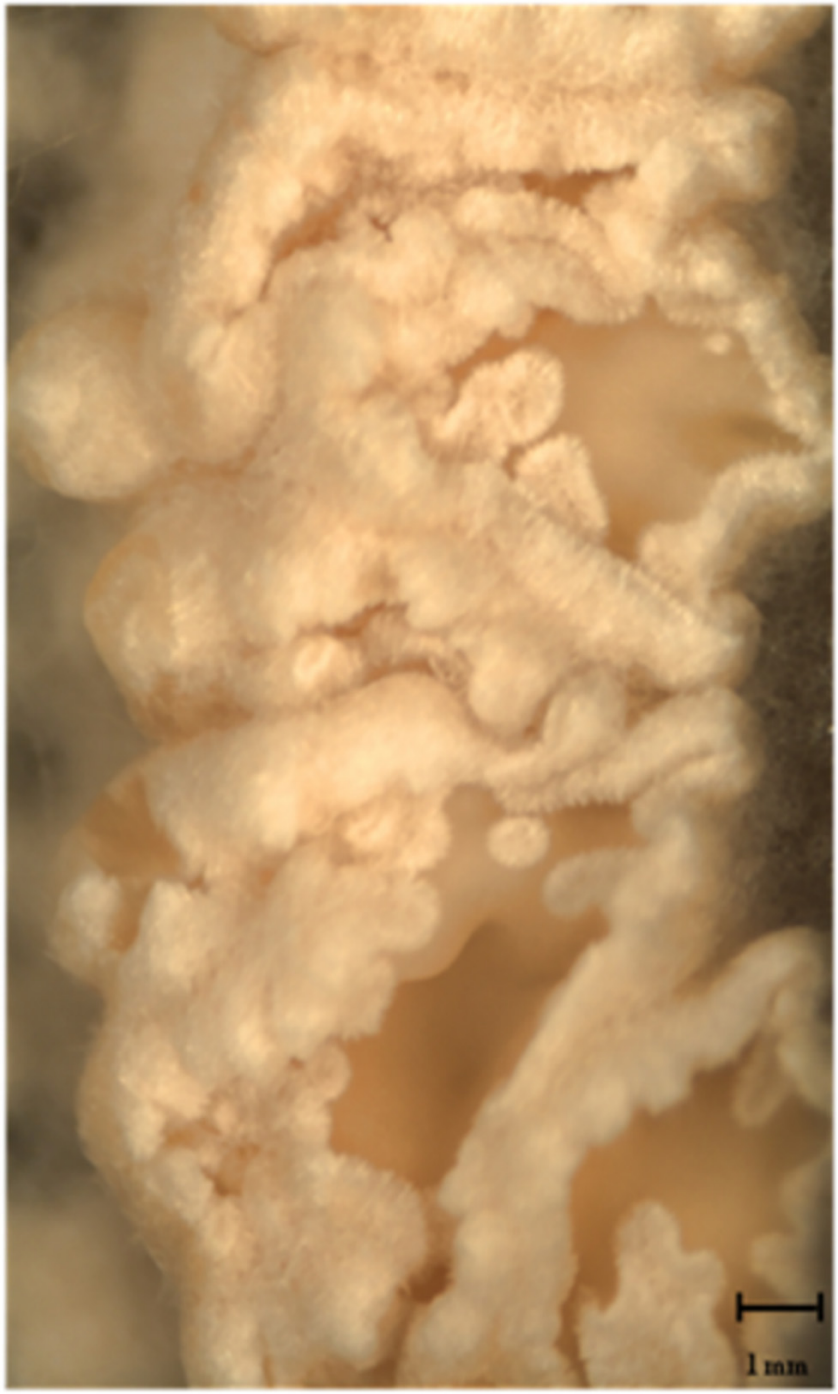
Fruiting bodies obtained from a mating between Δ*dhc1*_59 and wildtype strain T41. The lack of gill formation leads to a block in further development visible with the lack of fully developed fruitbodies.

**Fig 8:**
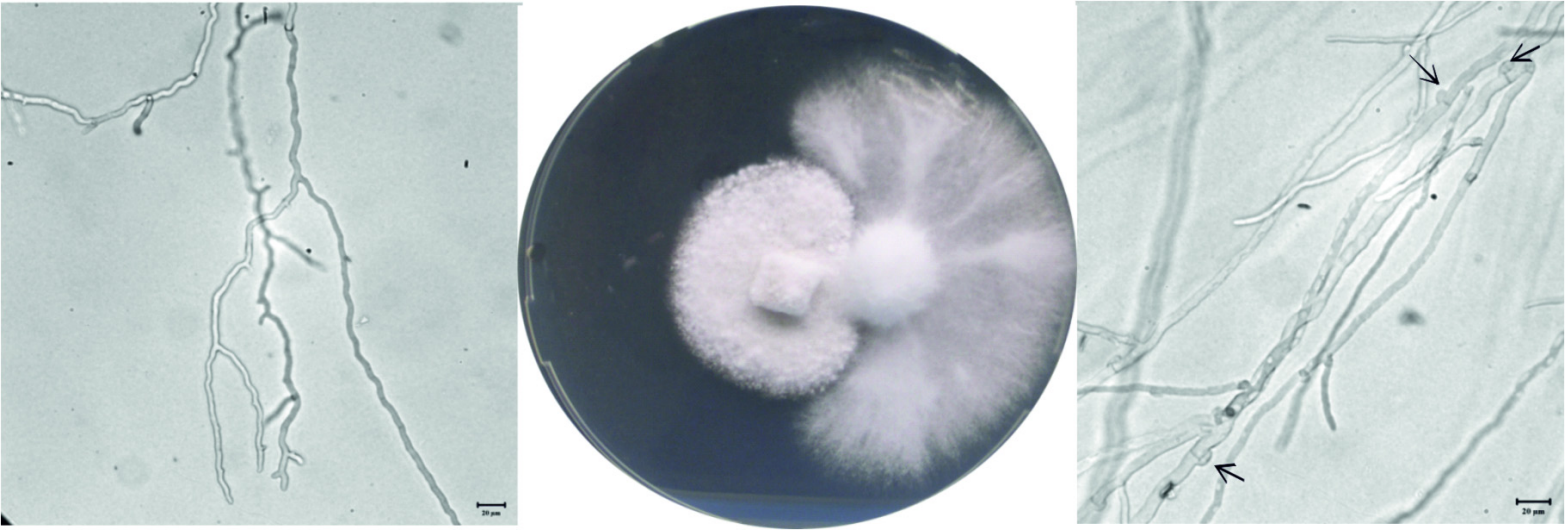
Compatible mating reaction between ∆*dhc2* (left) and wildtype strain 4–39 (right). Arrows indicate clamp cell formation only on the side of the wildtype strain. The wildtype, inoculated on the right, showed clamp formation which was lacking on the side of the mutant showing a unilateral donor phenotype of *dhc2* deletion.

We were able to obtain 1 Δ*dhc1* mutant and 7 ∆*dhc2* mutant strains with ∆*dhc2_4* and ∆*dhc2_5* used for further studies. The mutant strain ∆*dhc1*_59 produces a high amount of aerial mycelium. After five days of incubation at 30°C, the wildtype colony had covered an area of 9.5 ± 0.6 cm^2^, while the *dhc1* knock-out mutant only reached 3.27 ± 0.29 cm^2^. Thus, growth was reduced 2.91-fold. Cells of the mutant strain grow spherical. Analog to the reduced growth rate of the *dhc1* mutant, Δ*dhc2* colonies cover an area of 2.3 ± 0.6 cm^2^ which shows a four-fold reduced growth rate (Fig D in S1 File). The Δ*dhc2* strains show a dense colony structure with reduced aerial mycelium, contrasting to the fluffy structure of wildtype colonies (Fig 4). Furthermore, hyphae of ∆*dhc2* revealed a curly growth pattern. Additionally, a reduction in cell size associated with Δ*dhc1 and* ∆*dhc2* was visible microscopically. While wildtype strains show cell lengths varying from few to more than 270 μm, the cells in both mutants generally are shorter than 150 μm (Fig 5). Also, Δ*dhc1 and* ∆*dhc2* have a defect in nuclear positioning. Nuclei of wildtype strains are localized at the center of the cell, while in the mutants a displacement of nuclei was observed. In apical and subapical cells, the nucleus seems to lack preferential localization altogether (Fig 6). Strain ∆*dhc1*_59 shows normal mating behavior with a compatible mating partner in confrontation assays. Under optimal growth conditions also fruiting bodies can be formed by both partners. Nevertheless, the fruiting bodies formed on the mutant growth site are smaller and not fully opened after ten days of primordial appearance. No fully developed gills were seen in these fruiting bodies (Fig 7). In contrast, the ∆*dhc2* mutants show a defect in nuclear migration. In crosses, we observed that the mutant strain can donate nuclei to its mating partner, but ∆*dhc2* strains are unable to accept nuclei. A dikaryotic mycelium is seen only at the mating partner side of confrontation assays. Therefore, fruiting bodies appear only on the mating partner side. They do not show any changed phenotypes or differences in germination rate of the produced spores (Fig 8).

### Compensation of dynein function in viable deletion strains

In an attempt to identify differential functions for Dhc1 and Dhc2, we mated both deletion strains. Mating of the strains deleted for *dhc1* or *dhc2*, respectively, was performed to control for potential complementation. The resulting dikaryons showed normal mating behavior, although hyphae are still deformed. As fruiting bodies can be produced by both mating partners, the functions in meisosis, indeed, are complemented. The cellular functions are partly impaired by the resulting lower gene copy number.

To control for compensatory expression profiles in the deletion strain, RNAseq was performed. In ∆*dhc2*, a higher expression was found for two different kinesins, if compared to each of the wildtype strains. Kinesin-2 did show a 4.6- fold change, while kinesin-14 had a change of 9-fold in ∆*dhc2*. These results of higher expression were independently verified by quantitative real-time PCR. Indeed, both *kin14* and *kin2* could be shown to be induced in Δ*dhc2*. Thus, minus-end directed microtubule-dependent movement can be substituted in the motor domain knock-out strain by over-expression of kinesins known to be able to provide minus-end directed motor function. Indeed, all progeny contained a copy of *kin14* from the parent E6. Since the other parent, 12–43, did not yield a *kin14* amplicon, *kin14* was essential for survival of dynein deletion strains.

## Discussion

We could show that the heavy chain of cytoplasmic dynein of Basidiomycota is encoded by two separate proteins. While Dhc1 of *S*. *commune* encodes the N-terminal region of the heavy chain containing the dimerization domain, Dhc2 encodes a part of the tail region and the C-terminal motor and microtubule binding regions of homologous full length dynein. Still, the combined dynein heavy chain gene shows high similarity to dynein heavy chain genes in other fungi and other Eukaryotes.

Straube *et al*. [30] have shown that Dyn1 and Dyn2 of *Ustilago maydis* are part of a complex *in vivo*. It has to be assumed, supported by preliminary evidence of immunofluorescence (see S1 File), that both proteins interact to form the fully functional cytoplasmic dynein complex. The interaction between both parts is not governed by a specifically evolved protein domain at the break point, but rather seems to be facilitated through complex formation with the other proteins in the entire dynein machinery complex. Alternatively, the limited sequence similarities around the break point in the two proteins of basidiomycetes might be involved in an as of yet unknown protein-protein interaction.

Phylogenetic studies have revealed three independent splits during Basidiomycota evolution, with only Pucciniomycotina and Tremellomycetes featuring a single gene encoding the entire heavy chain of cytoplasmic dynein. The type of split is conserved in each of these groups, and reversions to a secondarily fused gene are not evident. Dynein heavy chain phylogeny largely reflects the currently accepted phylogeny of fungi despite the splitting of the gene during three independent events, and including the notoriously problematic Wallemiomycetes [53–55]. However, as in previous analyses, the relationship between Pucciniomycotina, Ustilaginomycotina and Agaricomycotina including Wallemiomycetes could not be resolved with clarity. Possibly due to long branch attraction effects, the extremely divergent Saccharomycotina and Taphrinomycotina dynein heavy chain genes are resolved as monophyletic clade, contrasting to current phylogenetic views [54, 55]. The obviously accelerated evolution within these groups might be attributed to their dominant yeast lifestyle, where dynein is involved in mitotic nuclear positioning in the bud. However, accelerated evolution is not evident in Ustilaginomycotina or Tremellomycetes, which also have yeast stages, albeit with reverse nuclear movement after mitosis in the bud. An exception to the generally well reflected phylogenetic relationships is seen with *Coprinopsis cinerea*, which in dynein phylogeny clusters within Polyporales instead of Agaricales.

The fact, that three independent split events occurred within the Basidiomycota, while no other eukaryote phylum has been shown to contain split dynein heavy chain genes, seems to imply a function exclusive to the basidiomycetes. Potentially, a moonlighting function for one of the two gene products might be realized. The *dhc1* and *dhc2* deletion is viable, in contrast to *U*. *maydis* dynein heavy chain genes [30]. Thus, different proteins, most likely kinesin-14 homologs, are able to take over microtubular minus-end directed transport. Kinesin-14 is minus-end directed, and required for assembly of the mitotic spindle [56, 57]. In *Drosophila melanogaster*, kinesin-14 is necessary for spindle assembly and spindle organization [58]. Dynactin interactions of kinesin-5 have been shown to also provide minus-end directed movement on microtubules [59]. Kinesin-5 is a slow motor which regulates microtubule-microtubule interaction during mitosis and slows down the separation rate of half-spindles during mitosis in vertebrates [60]. As summarized in Ferenz, *et al*. [61], kinesin-5 has been identified as an important motor protein in mitosis in several model organisms like *Saccharomyces cerevisiae*, *Drosophila* and *Arabidopsis*, where it is required for spindle assembly. In fungi, kinesin-5 is necessary for keeping up the bipolarity of the mitotic spindle, as well as spindle elongation in anaphase. We were able to show a higher expression of kinesin-2 (homologues to kinesin-5 of *Aspergillus nidulans*) and kinesin-14 in ∆*dhc2*. This fact provides an explanation for the viability of the mutant and hints at minus-end directed movement known to be exerted by kinesin-14. A similar result seems to be probable for the Δ*dhc1* strain but must be verified by transcriptome analysis in the future.

The phenotypes of the *S*. *commune* dynein mutants are comparable to those described for the filamentous Ascomycota *Aspergillus nidulans* and *Neurospora crassa*. Both show retarded growth and a defect in nuclear migration and positioning [62, 63]. Dynein interaction with Lis1 (related to the human disease lissencephaly) is essential for its function in nuclear and spindle positioning in fungi. Lis1 exclusively is able to bind to the first AAA module of the dynein heavy chain to activate dynein motility [59, 64, 65]. In ∆*dhc2*, the first AAA module is missing and thus, Lis1 interaction is impaired. This may well lead to the altered nuclear positioning observed in ∆*dhc2* and subsequently to shorter cell lengths, as septa are formed at the place of the mitotic spindle in Basidiomycota. As the Δ*dhc1* strains shows the same phenotypical changes, an overlapping function of both genes seems likely, mainly functioning in maintaining cell shape, distribution of nuclei and positioning of the nucleus in every cell. Additionally, *dhc2* seems to function in nuclear migration, whereas *dhc1* has no influence on this process.

With the Basidiomycota, the dikaryotic stage and the mating associated long-term nuclear migration are specific for their life style. The deletion of *dhc1* mainly resulted in meiotic deficiency which indicated a function in spindle formation. With the deletion of *dhc2*, however, nuclear migration was impaired and a phenotype was visible with selectively donating nuclei in a mating. Together, this might be indicative of one complex formed between Dhc1 and Dhc2 involved in cellular functions including meiosis. Another protein complex with one or several proteins would then replace Dhc1 in nuclear migration. In addition, Dhc2 seemed to be involved in tip growth (see Fig 8, left side distorted growth orientation of hyphae). The partly overlapping functions of both proteins would constitute a selection pressure towards seperate proteins to allow for differentiation of functions within nuclear distribution. Our investigation of Basidiomycete dynein heavy chain yielded a new example for separate routes in evolution, even for a highly conserved and structurally important protein complex like dynein. The phenotypic analyses now open a route to screen for suppressors, which will allow unraveling of networks for dynein interaction, and possibly including functions of moonlighting not seen with other eukaryotes.

## Supporting Information

S1 File Data sources for phylogenetic analyses, accessed December 2011. Representatives of Agaricomycotina are indicated as classes; for Wallemiomycetes, no subphylum is assigned (Table A).**Conservation of genomic localization for dhc1 and dhc2 with respect to linkage to the mating type A locus in hymenomycetes.** *S*. *commune* as well a *Fomitiporia mediterranea*, *Phanorachaete chrysosporium*, *Ganoderma lucidum*, *Auricularia delicate*, *Pleurotus ostreatus* and *Wolfiporia cocos* are shown, data were retrieved from genome sequences in public databases (Fig A). **Immunofluorescence co-localization.** Dhc1 (A, FITC labeled) and Dhc2 (B, Rhodamin labeled), combined with nuclar stain (C, DAPI stain) and micrograph (D), superimposed picture (E) and enlarged view indicated in E (F) (Fig B). **Dhc1 aggregation is independent from microtubules in accordance with a storage independent from Dhc2**. Microtubule (in green), Dhc1 (in red) and DNA staining (in blue) (Fig C). **No obvious alterations of the microtubular cytoskeleton is visible.** Microtubule (in green) and DNA staining (in blue) in a *Δdhc2* mutant (Fig D).
(PDF)
