# Supplementary material for: Dynein Heavy Chain, Encoded by Two Genes in Agaricomycetes, Is Required for Nuclear Migration in Schizophyllum commune
Source: PLoS One. 2015 Aug 18;10(8):e0135616. doi: 10.1371/journal.pone.0135616 (PMC4540427; doi:10.1371/journal.pone.0135616)
Supplement: S1 File — Representatives of Agaricomycotina are indicated as classes; for Wallemiomycetes, no subphylum is assigned (Table A).Conservation of genomic localization for dhc1 and dhc2 with respect to linkage to the mating type A locus in hymenomycetes. S. commune as well a Fomitiporia mediterranea, Phanorachaete chrysosporium, Ganoderma lucidum, Auricularia delicate, Pleurotus ostreatus and Wolfiporia cocos are shown, data were retrieved from genome sequences in public databases (Fig A). Immunofluorescence co-localization. Dhc1 (A, FITC labeled) and Dhc2 (B, Rhodamin labeled), combined with nuclar stain (C, DAPI stain) and micrograph (D), superimposed picture (E) and enlarged view indicated in E (F) (Fig B). Dhc1 aggregation is independent from microtubules in accordance with a storage independent from Dhc2. Microtubule (in green), Dhc1 (in red) and DNA staining (in blue) (Fig C). No obvious alterations of the microtubular cytoskeleton is visible. Microtubule (in green) and DNA staining (in blue) in a Δdhc2 mutant (Fig D). (PDF) [file pone.0135616.s001.pdf]

S1 File

Supplementary Table A. Data sources for phylogenetic analyses, accessed December 2011. Representatives of Agaricomycotina are indicated as classes; for Wallemiomycetes, no subphylum is assigned.

species	phylum	subphylum/class	database	accession number(s)
Agaricus bisporus var bisporus H97	Basidiomycota	Agaricomycetes	JGI	188324<br>214780
Agaricus bisporus var burnetti JB137-S8	Basidiomycota	Agaricomycetes	JGI	80770<br>50984
Ashbya gossypii	Ascomycota	Saccharomycotina	NCBI	NP983660
Aspergillus fumigatus	Ascomycota	Pezizomycotina	NCBI	XP753470
Aspergillus nidulans	Ascomycota	Pezizomycotina	NCBI	XP657722
Auriculara delicata SS-5 v 1.0	Basidiomycota	Agaricomycetes	JGI	78165<br>179093
Bjerkandera adusta v 1.0	Basidiomycota	Agaricomycetes	JGI	164640<br>210459
Botryobasidium botryosum_v 1.0	Basidiomycota	Agaricomycetes	JGI	26434<br>26427
Candida albicans	Ascomycota	Saccharomycotina	NCBI	XP723061
Coniophora puteana v 1.0	Basidiomycota	Agaricomycetes	JGI	114255<br>133816
Coprinopis cinereus Okayama 7#130	Basidiomycota	Agaricomycetes	JGI	11129<br>3276
Cryptococcus bacillosporus	Basidiomycota	Tremellomycetes	Broad Institute	CNBF3870
Cryptococcus gatti WM276	Basidiomycota	Tremellomycetes	NCBI	XP003194894
Cryptococcus neoformans var neoformans JEC21	Basidiomycota	Tremellomycetes	NCBI	XP571532
Dacryopinax sp. DJM 731 SSP-1 v 1.0	Basidiomycota	Dacryomycetes	JGI	67652<br>116759
Dichomitus squalens v 1.0	Basidiomycota	Agaricomycetes	JGI	172670<br>182524
Fomitiporia mediterranea v 1.0	Basidiomycota	Agaricomycetes	JGI	106792<br>121816
Fomitopsis pinicola SS1 v 1.0	Basidiomycota	Agaricomycetes	JGI	140971<br>150781
Ganoderma sp 10597 SS1 v 1.0	Basidiomycota	Agaricomycetes	JGI	113078<br>140853
Gloeophyllum trabeum v 1.0	Basidiomycota	Agaricomycetes	JGI	69644<br>135602
Gymnopus luxurians v 1.0	Basidiomycota	Agaricomycetes	JGI	961840<br>234850
Heterobasidion annosum v 2.0	Basidiomycota	Agaricomycetes	JGI	157884<br>167275
Homo sapiens	Mammalia	Hominidae	NCBI	NP001267
Hypholoma sublateritium v 1.0	Basidiomycota	Agaricomycetes	JGI	42741<br>509615
Jaapia argyllacea v 1.0	Basidiomycota	Agaricomycetes	JGI	140422<br>37924
Laccaria bicolor v 2.0	Basidiomycota	Agaricomycetes	JGI	385191<br>300914
Magnaporthe oryzae 70 50	Ascomycota	Pezizomycotina	NCBI	EDK04833
Malassezia globosa	Basidiomycota	Ustilaginomycotina	JGI	1036<br>1033
Melampsora laricis populina v 1.0	Basidiomycota	Pucciniomycotina	JGI	46877
Moniliophthora perniciosa	Basidiomycota	Agaricomycetes		(personal communication J. Mondego, Brazil)
Mucor circinelloides	Mucoromycota	Mucoromycotina	JGI	90435
Mus musculus	Mammalia	Rodentia	NCBI	NP084514
Nectria haematococca	Ascomycota	Pezizomycotina	NCBI	EEU40393
Neurospora crassa	Ascomycota	Pezizomycotina	NCBI	XP962616
Paxillus involutus ATCC	Basidiomycota	Agaricomycetes	JGI	169564

200175				5609
Phanerochaete carnosa HHB-10118-Sp v 1.0	Basidiomycota	Agaricomycetes	JGI	256688<br>168059
Phanerochaete chrysosporium v. 2.0	Basidiomycota	Agaricomycetes	JGI	7743<br>7621
Phlebia brevispora HHB-7030 SS6 v 1.0	Basidiomycota	Agaricomycetes	JGI	77449<br>138126
Phlebiopsis gigantea v 1.0	Basidiomycota	Agaricomycetes	JGI	34940<br>29508
Pichia pastoris GS115	Ascomycota	Saccharomycotina	NCBI	XP002491674
Piriformospora indica	Basidiomycota	Agaricomycetes	MIPS	PIIN_08066<br>PIIN_03421
Pleurotus ostreatus PC9 v 1.0	Basidiomycota	Agaricomycetes	JGI	90323<br>85298
Pleurotus ostreatus PC15 v 2.0	Basidiomycota	Agaricomycetes	JGI	1074656<br>49217
Puccinia graminis f sp tritici CRL 75 36 700 3	Basidiomycota	Pucciniomycotina	NCBI	XP0033294
Punctularia strigosozonata v 1.0	Basidiomycota	Agaricomycetes	JGI	109520<br>139986
Rhizopus oryzae RA 99 880	Mucoromycota	Mucoromycotina	Broad Institute	RO3G06230
Rhodotorula glutinis	Basidiomycota	Pucciniomycotina	NCBI	EGU12573
Rhodotorula graminis strain WP1 v 1.0	Basidiomycota	Pucciniomycotina	JGI	34525
Saccharomyces cerevisiae	Ascomycota	Saccharomycotina	NCBI	EDN59957
Schizophyllum commune v 1.0	Basidiomycota	Agaricomycetes	JGI	65157<br>65089
Schizosaccharomyces pombe 972h	Ascomycota	Taphrinomycotina	NCBI	XP001713108
Serpula lacrymans S7 3 v 2.0	Basidiomycota	Agaricomycetes	JGI	97057<br>158538
Serpula lacrymans S7 9 v 1.0	Basidiomycota	Agaricomycetes	JGI	358531<br>445906
Sporisorium reilianum	Basidiomycota	Ustilaginomycotina	MIPS	sr13295<br>sr15257
Sporobolomyces roseus v 1.0	Basidiomycota	Pucciniomycotina	JGI	21679
Stereum hirsutum FP- 91666 SS1 v 1.0	Basidiomycota	Agaricomycetes	JGI	143406<br>151431
Trametes versicolor v 1.0	Basidiomycota	Agaricomycetes	JGI	111059
Tremella mesenterica Fries v 1.0	Basidiomycota	Tremellomycetes	JGI	62339
Tuber melanosporum	Ascomycota	Pezizomycotina	INRA	GSTUMT00000794001
Ustilago maydis	Basidiomycota	Ustilaginomycotina	NCBI	AAK91759<br>AAK91760
Wallemia sebi v 1.0	Basidiomycota	Wallemiomycetes	JGI	59093<br>67017
Wolfiporia cocos MD-104 SS10 v 1.0	Basidiomycota	Agaricomycetes	JGI	93003<br>159953

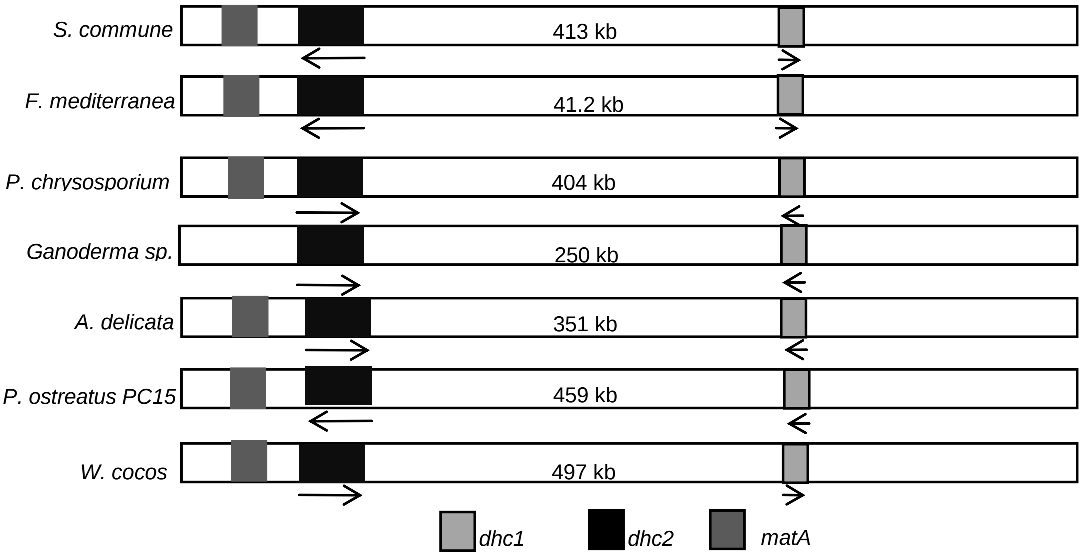

Supplementary Fig. A. Conservation of genomic localization for *dhc1* and *dhc2* with respect to linkage to the mating type *A* locus in hymenomycetes. *S. commune* as well a *Fomitiporia mediterranea, Phanorachaete chrysosporium, Ganoderma lucidum, Auricularia delicate, Pleurotus ostreatus* and *Wolfiporia cocos* are shown, data were retrieved from genome sequences in public databases.

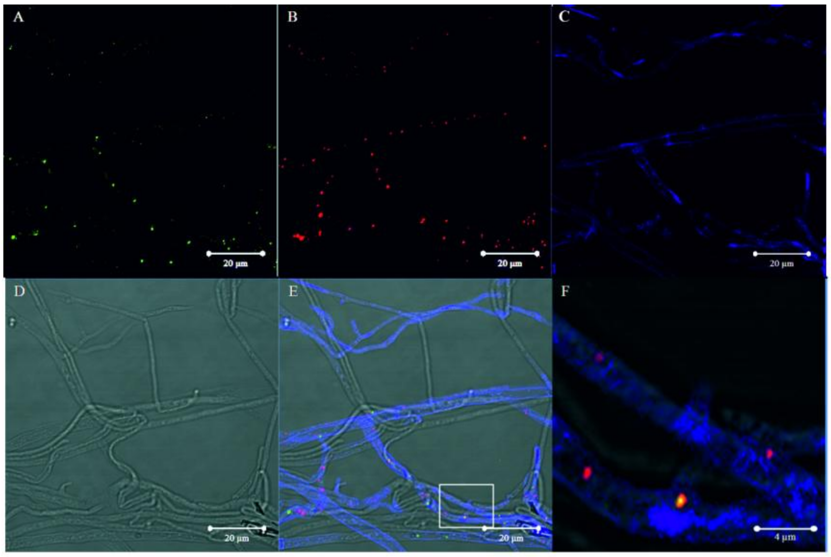

Supplementary Fig. B. Immunofluorescence co-localization. Dhc1 (A, FITC labeled in green) and Dhc2 (B, Rhodamin labeled in red), combined with nuclar stain (C, DAPI stain in blue) and micrograph (D), superimposed picture (E) and enlarged view indicated in E (F).

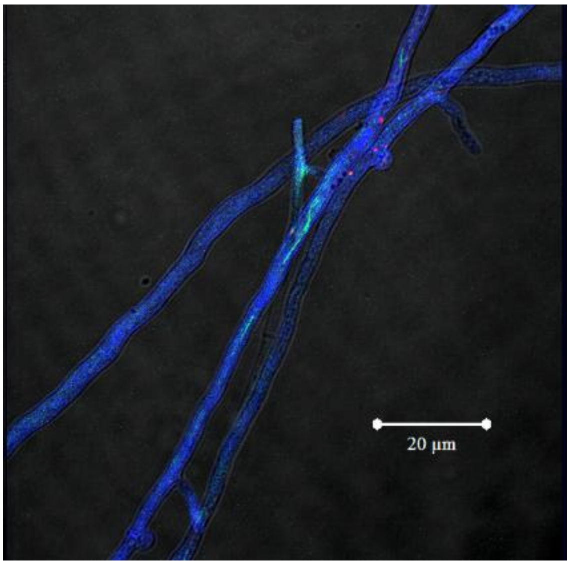

Supplementary Fig. C. Dhc1 aggregation to be independent from microtubules in accordance with a storage independent from Dhc2. Microtubule (in green), Dhc1 (in red) and DNA staining (in blue).

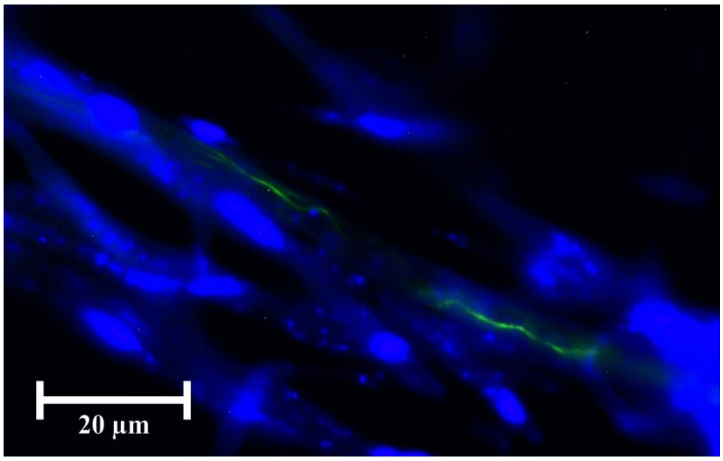

Supplementary Fig. D. No obvious alterations of the microtubular cytoskeleton is visible. Microtubule (in green) and DNA staining (in blue) in a Δ*dhc2* mutant.
